# Delayed Neurologic Deficit due to Foraminal Stenosis following Osteoporotic Late Collapse of a Lumbar Spine Vertebral Body

**DOI:** 10.1155/2013/682075

**Published:** 2013-03-04

**Authors:** Yu Sasaki, Yasuchika Aoki, Arata Nakajima, Yoshifumi Shibata, Masato Sonobe, Kazuhisa Takahashi, Seiji Ohtori, Koichi Nakagawa

**Affiliations:** ^1^Department of Orthopedic Surgery, Toho University Sakura Medical Center, 564-1 Shimoshizu, Sakura, Chiba 285-8741, Japan; ^2^Department of Orthopedic Surgery, Graduate School of Medicine, Chiba University, 1-8-1 Inohana, Chuo-ku, Chiba 260-8670, Japan

## Abstract

We report an 85-year-old woman with an L3 vertebral body fracture who presented with back pain, bilateral leg pain, and weakness after four months of conservative treatment. Because of unstable pseudoarthrosis, the L3 vertebral body collapsed in the standing position and the L3 nerve root was compressed. The indicated surgery decompressed the L3-L4 foramen and fused the unstable segment. The back pain and neurologic symptoms improved significantly following surgery. We propose that delayed neurologic deficit following an osteoporotic fracture of the lumbar body may be caused not only by retropulsion of vertebral body fragments with significant canal compromise, but also by foraminal stenosis with the late collapse of the vertebral fracture. This new pathomechanism for delayed neurologic deficit has not been previously described. If a collapse takes place in the caudal part of the vertebral body below the base of the pedicle, spine surgeons should be aware of the possibility of foraminal stenosis.

## 1. Introduction

Osteoporotic vertebral compression fracture occurs most often in postmenopausal women age 60 years and older, after minimal trauma or spontaneously [1]. It usually causes acute symptoms associated with localized pain of variable intensity at the fracture site. The most serious complication of this fracture is delayed neurologic deficit, usually due to the retropulsion of vertebral body fragments with significant canal compromise, pseudoarthrosis, or local kyphosis [2, 3]. Shikata et al. found that 12.1% of these fractures required surgery because of neurological sequelae. Controversy exists over whether insufficiency fractures of the spine can lead to collapse, resulting in the formation of local kyphosis. A patient with an L3 osteoporotic vertebral fracture recently presented with delayed neurologic deficit. In this patient, the L3 fracture failed to accomplish bony fusion and developed pseudoarthrosis; however, no retropulsion of bony fragments was found. The pathogenesis of the neurologic deficit could not be diagnosed and the patient was referred to our department. We carefully assessed symptoms and radiological findings and concluded that the neurologic deficit was caused by bilateral L3-L4 intervertebral foraminal stenosis. To our knowledge, there is no previous report describing delayed neurologic deficit caused by foraminal stenosis. In this report we present the clinical and radiological findings and discuss the pathomechanism of the neurologic deficit in this case.

## 2. Case Report

**Figure 1:**
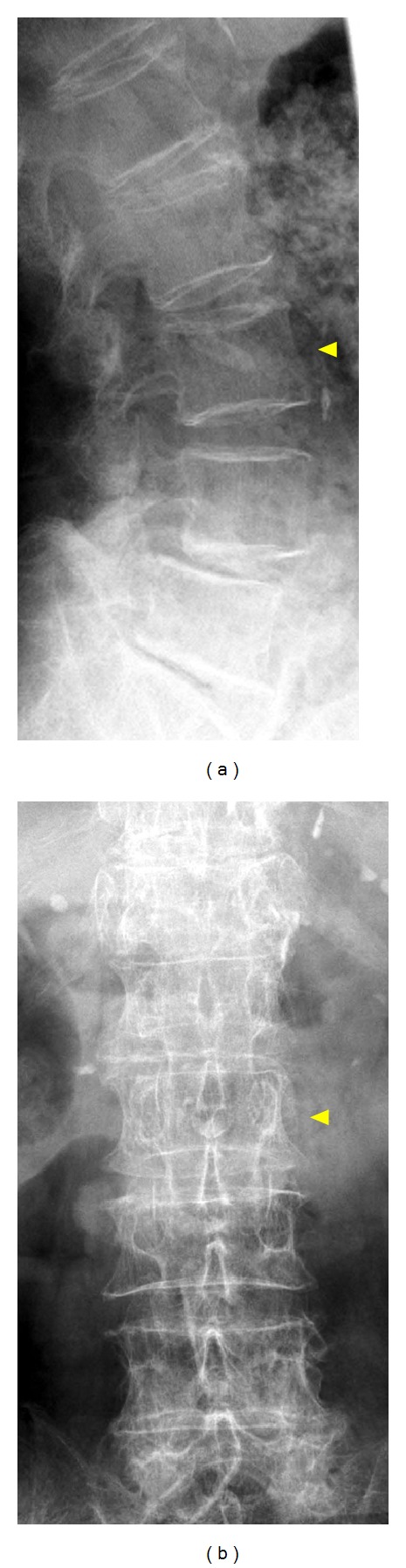
Anteroposterior (a) and lateral (b) radiographs of the lumbar spine of an 85-year-old woman taken immediately after a simple fall, when the fracture in the L3 body (arrowhead) was not obvious.

**Figure 2:**
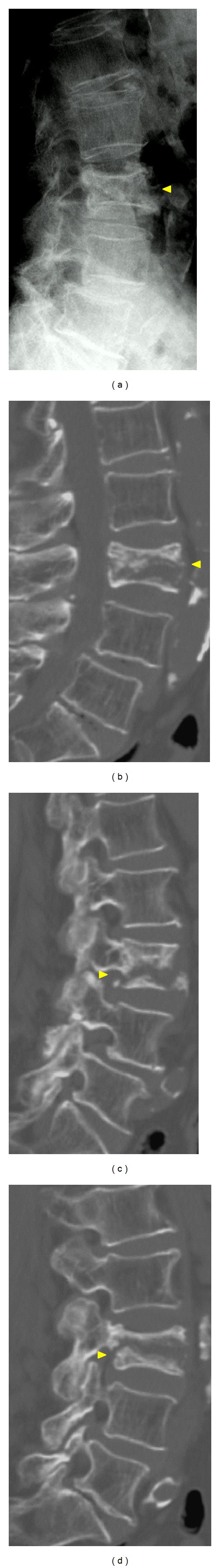
The 85-year-old woman with an L3 vertebral body fracture, detected two weeks after the initial onset by magnetic resonance imaging, presented with back pain, bilateral leg pain, and weakness after four months of conservative treatment. The lateral radiograph (a) in supine position showed the collapse of the L3 vertebral body compared with the initial radiographs. Reconstructed computed tomography (CT) taken in the supine position (b) shows an osteoporotic vertebral collapse of L3 (arrowhead) with an interior bony defect. Parasagittal images of the CT scan show the collapsed vertebral body on both sides (c) and (d). Note the fracture lines from the undersurface of the L3 pedicle to the anterior side of the L3 vertebral body (arrowhead).

**Figure 3:**
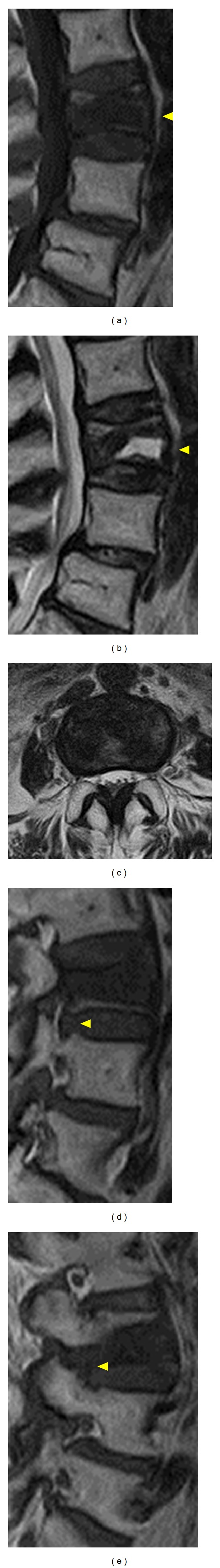
T1- (a) and T2-weighted sagittal (b) magnetic resonance (MR) imaging performed after four months of conservative treatment of an 85-year-old woman with an L3 vertebral body fracture shows fluid collection in the L3 vertebral body, indicating L3 pseudoarthrosis with no spinal canal stenosis at the L3 level (arrowhead). T2-weighted axial MR image (c) shows spinal canal stenosis at the L3-L4 level with facet and ligamentum flavum hypertrophy. Parasagittal T1-weighted (d: Left side e: Right side) MR imaging showing foraminal stenosis with circumferential loss of perineural fat signal at the L3-L4 level, in contrast with the intense fat signal at the adjacent foraminal levels.

Following a simple fall at home, an 85-year-old woman presented with back pain as a chief complaint and was referred to a hospital. Magnetic resonance imaging (MRI) taken two weeks after the initial onset showed a fresh L3 vertebral fracture although plain radiograms had failed to detect the fracture (Figure 1). She initially had back pain without lower extremity symptoms and was treated conservatively. One month after the initial onset, bilateral leg pain appeared and gradually worsened. At four months after the initial onset she was referred to our hospital because the pain in her back and bilateral leg pain worsened in a standing or sitting position, decreasing during bed rest. The radicular leg pain was so severe that it was impossible for her to sit in a wheelchair. Physical examination revealed that bilateral radicular pain and numbness affected the bilateral anterior thigh, with MRC grade 3-4/5 weakness of the iliopsoas, and quadriceps femoris in both legs. Plain radiographs showed the L3 vertebral body more collapsed compared with the initial radiographs, and computed tomography (CT) scans showed a bony defect inside the fractured vertebral body (Figure 2). MRI showed fluid collection in the fractured L3 vertebral body, suggestive of pseudoarthrosis of the L3 vertebral body. There was no retropulsion of bony fragments into the spinal canal. Spinal canal stenosis due to L4 spondylolisthesis was seen at the L4-L5 level (Figure 3). However, these findings did not fully explain the patient's neurological status, which seemed to be caused by L3 (or L4) nerve root involvement. Importantly, bilateral foraminal stenosis at L3-L4 was present on MRI (Figure 3), which may produce L3 nerve root compression. 

**Figure 4:**
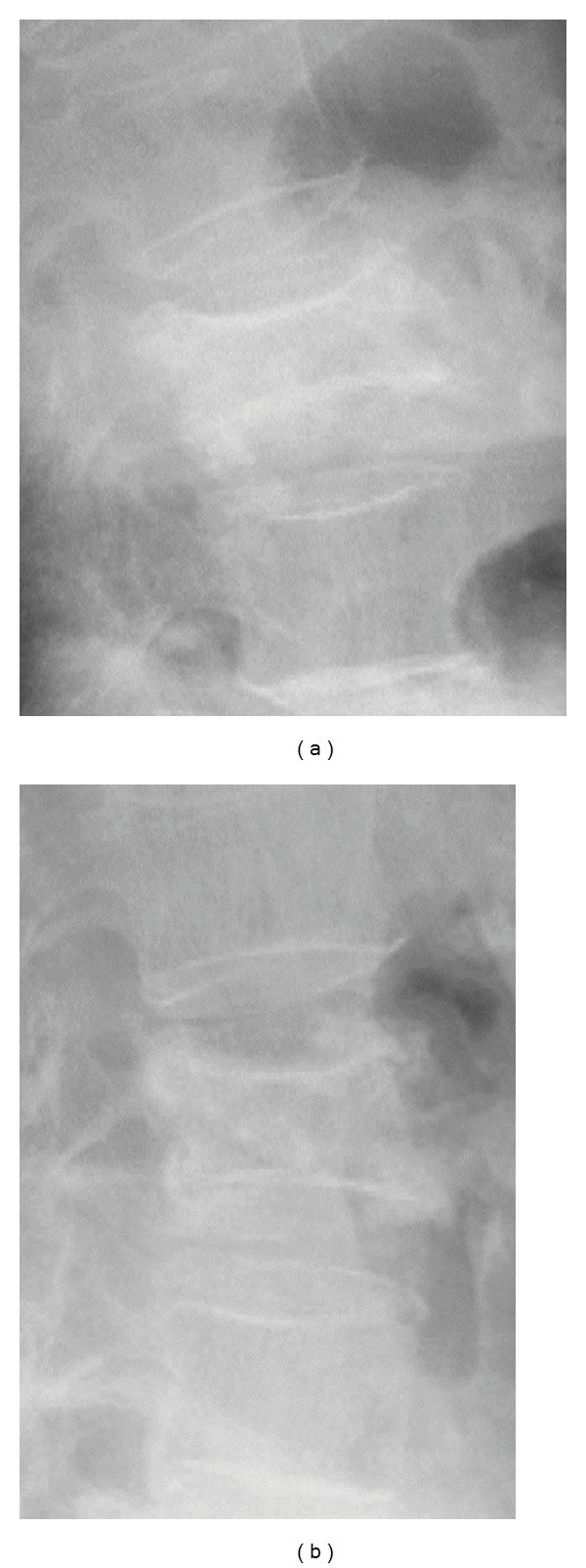
Lateral radiographs (a) taken in standing and (b) supine positions after four months of conservative treatment of an 85-year-old woman with an L3 vertebral body fracture show the L3 vertebral body more collapsed in the standing (a) than in the supine position (b).

**Figure 5:**
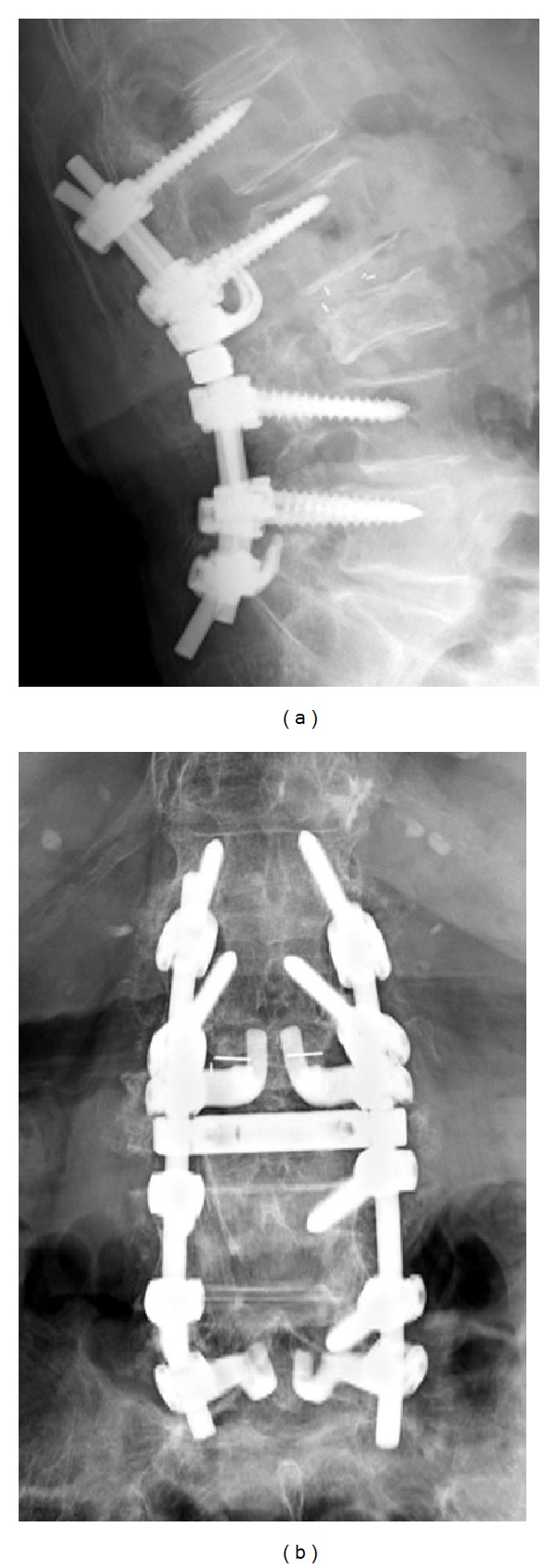
Posterior decompression and fusion surgery was performed on the 85-year-old woman with an L3 vertebral body fracture. Postoperative anteroposterior (a) and lateral (b) radiographs show appropriate positioning of intervertebral cages and pedicle screws.

Upon the reexamination of the plain radiographs in standing and supine positions, we saw that the caudal part of the L3 vertebral body collapsed in the standing position, resulting in the exacerbation of the L3-L4 foraminal stenosis (Figure 4). This led us to conclude that the patient's neurologic deficit was due to L3-L4 foraminal stenosis although a more detailed examination, such as electromyography, was not performed. Posterior decompression and fusion surgery decompressed the L3-L4 foramen and fused the unstable segment. The cranial half of the L3 lamina was removed and served as a local bone graft. The L3 pedicle was subtracted and the cranial part of the L3 vertebral body and L2-L3 disc were resected. Two intervertebral cages (PEEK OIC Cages: Stryker, Allendale, NJ, USA) with local and iliac bone grafts were placed between the L2 endplate and the remaining L3 vertebral body (Figure 5).

A pedicle screw system (Legacy: Medtronic Sofamor Danek, Memphis, TN, USA) was utilized, placing two screws above and two screws below the site, and posterolateral fusion was performed. A dramatic neurological improvement occurred following the surgery. By the 7th postoperative day, the patient could walk using parallel bars with a thoracolumbosacral orthosis for external support. No postoperative complications developed. Two months after surgery, the leg pain was completely gone, the muscle weakness fully recovered, and the patient could walk without support. At the final followup 18 months postoperatively she had no pathological symptoms related to spinal fracture. 

## 3. Discussion

When treating patients with degenerative lumbar spinal disease, intervertebral foraminal stenosis tends to be overlooked because a diagnostic method is not well established. Because of the difficulty in making a correct diagnosis, this condition may result in failed back surgery syndrome, producing continued postoperative pain [4]. It is important to be aware of intervertebral foramina when treating patients with degenerative lumbar spinal diseases presenting with radicular leg pain. Some recent reports in Japan have proposed the possibility of foraminal stenosis after vertebral fracture [5, 6]. However, there are no reports describing delayed neurologic deficit due to foraminal stenosis in international journals. Hasegawa et al. described the relationship between foraminal stenosis of the lumbar spine and posterior disc heights [7]. In our case, the foraminal stenosis was not related to lowered disc height and may have been caused by collapse of the caudal part of the vertebral body. Hasegawa et al. reported that a foraminal height lower than 15 mm is a risk factor for nerve root involvement [7]. In our patient, the foraminal height was measured at 16 mm in the supine position, but only 10 mm in the standing position (Figure 4), showing that foraminal height decreased in the standing position. This finding supports our diagnosis and explains why the lower extremity pain dramatically worsened when standing. Inufusa et al., using CT and cryomicrotome analyses, have shown that lumbar flexion caused a 12% increase in the foraminal area, whereas extension produced a 15% decrease [8]. This concept of dynamic foraminal stenosis supports our diagnosis. Generally, foraminal stenosis is classified into anteroposterior stenosis “transverse stenosis” and craniocaudal stenosis “vertical stenosis” [9]. Protrusion of the posterior wall of the vertebral body and facet hypertrophy can cause anteroposterior compression. Reduced disc height is a cause of craniocaudal compression in lumbar degenerative disease; however, reduced vertebral body height under the upper pedicle can cause craniocaudal compression in a fractured spine. In our patient, a large bony defect in the L3 body may have collapsed in the standing position, resulting in craniocaudal compression of the intervertebral foramen. This is a vertical type of foraminal stenosis because the undersurface of the L3 pedicle compresses the exiting nerve root against the upper surface of the L4 pedicle when load-bearing [9]. Thus, if a bony defect is detected at the caudal side of vertebral body between the pedicle and the inferior endplate, foraminal stenosis should be considered as a cause of nerve root compression when symptoms and physical findings suggest neurological compression. From these findings, we postulate that the primary mechanism for our patient's delayed neurologic deficit was bilateral foraminal stenosis. 

Our study has several limitations to exclude the possibility of other pathologies causing the observed symptoms. First, the surgery should be limited to decompression of the L3-L4 intervertebral foramen to determine the pathogenesis. However, if foraminal stenosis occurs in a patient with pseudoarthrosis, it may be risky to treat the patient with decompression surgery without fusion. Also, as mentioned above, more detailed examinations, such as electromyography, were not performed. It is generally recognized, however, that a definite diagnosis of foraminal stenosis is difficult even with thorough examinations and should be based on a comprehensive evaluation [10]. Importantly, in our patient, no retropulsion of bony fragment was present, and the cause of the L3 nerve root involvement was not determined until she was under our care. It would have been difficult for us to make a surgical decision if the foraminal stenosis had been overlooked. From the preoperative findings, we believe that the patient's symptoms were not caused by L4-L5 spinal canal stenosis. First, the patient's symptoms gradually worsened, related to the development of the L3 late collapse; second, the symptoms cannot be explained by L5 nerve root involvement; third, the symptoms were dramatically exacerbated in the standing position.

That our preoperative assessment was accurate is shown by the favorable surgical result. Previous papers describe the usual causes of delayed neurologic deficit as retropulsion of bony fragments and local kyphosis [2, 3]. We propose the importance of foraminal stenosis caused by late collapse of vertebral fracture as a pathomechanism for delayed neurological deficit. In conclusion, spine surgeons should be aware of the possibility of foraminal stenosis in cases of pseudoarthrosis, particularly if the collapse takes place in the caudal part of the vertebral body below the base of the pedicle.
